# Integrative Single-Cell RNA-Seq and ATAC-Seq Analysis of Mesenchymal Stem/Stromal Cells Derived from Human Placenta

**DOI:** 10.3389/fcell.2022.836887

**Published:** 2022-04-05

**Authors:** Jinlu Li, Quanlei Wang, Yanru An, Xiaoyan Chen, Yanan Xing, Qiuting Deng, Zelong Li, Shengpeng Wang, Xi Dai, Ning Liang, Yong Hou, Huanming Yang, Zhouchun Shang

**Affiliations:** ^1^ College of Life Sciences, University of Chinese Academy of Sciences, Beijing, China; ^2^ BGI-Shenzhen, Shenzhen, China; ^3^ Key Laboratory of Regenerative Medicine of Ministry of Education, Biology Postdoctoral Research Station, Jinan University, Guangzhou, China; ^4^ James D. Watson Institute of Genome Sciences, Hangzhou, China; ^5^ BGI College, Northwest University, Xi’an, China

**Keywords:** single-cell RNA-seq, single-cell ATAC-seq, human placenta, cell heterogeneity, mesenchymal stem cells, immunomodulatory-potential

## Abstract

Mesenchymal stem/stromal cells derived from placenta (PMSCs) are an attractive source for regenerative medicine because of their multidifferentiation potential and immunomodulatory capabilities. However, the cellular and molecular heterogeneity of PMSCs has not been fully characterized. Here, we applied single-cell RNA sequencing (scRNA-seq) and assay for transposase-accessible chromatin sequencing (scATAC-seq) techniques to cultured PMSCs from human full-term placenta. Based on the inferred characteristics of cell clusters, we identify several distinct subsets of PMSCs with specific characteristics, including immunomodulatory-potential and highly proliferative cell states. Furthermore, integrative analysis of gene expression and chromatin accessibility showed a clearer chromatin accessibility signature than those at the transcriptional level on immunomodulatory-related genes. Cell cycle gene-related heterogeneity can be more easily distinguished at the transcriptional than the chromatin accessibility level in PMSCs. We further reveal putative subset-specific *cis*-regulatory elements regulating the expression of immunomodulatory- and proliferation-related genes in the immunomodulatory-potential and proliferative subpopulations, respectively. Moreover, we infer a novel transcription factor *PRDM1*, which might play a crucial role in maintaining immunomodulatory capability by activating *PRDM1*-regulon loop. Collectively, our study first provides a comprehensive and integrative view of the transcriptomic and epigenomic features of PMSCs, which paves the way for a deeper understanding of cellular heterogeneity and offers fundamental biological insight of PMSC subset-based cell therapy.

## Introduction

Mesenchymal stem/stromal cells (MSCs) are promising cell candidates for regenerative medicine and cell therapy owing to their differentiation potential and cytokine regulation capability. For multidirectional differentiation, MSCs can differentiate into mesodermal lineage cells, such as adipocytes, osteocytes, and chondrocytes, as well as other cell lineages, such as endodermic and neuroectodermic cells (Petersen et al., 1999; Macias et al., 2010). In addition, MSCs have broad anti-inflammatory and immunomodulation properties as they can secrete several cytokines, such as growth factors or anti-inflammatory mediators, to modulate immune cell populations (Shi et al., 2018; Pittenger et al., 2019). Substantial progress has been made in the exploration of MSCs in regenerative and immunomodulation treatment (Rodríguez-Fuentes et al., 2021). Recently published phase I/II clinical trials about MSCs infusion in COVID-19 patients show that MSC infusion is safe and well-tolerated (Guo Z. et al., 2020; Feng et al., 2020; Meng et al., 2020), and it shows an efficient improvement trend in patients and reduces the mortality rate (Shu et al., 2020). Meanwhile, patients with MSC infusion show a faster recovery and significantly elevated lymphocyte counts (Guo Z. et al., 2020; Shu et al., 2020) and improvements in CD4^+^ T cells, CD8^+^ T cells, and NK cell counts (Feng et al., 2020), indicating significant immunomodulation effects of MSCs.

MSCs can be isolated from various tissues (da Silva Meirelles et al., 2006), such as bone marrow (Prockop, 1997), adipose tissue (Zuk et al., 2003), umbilical cord (Romanov et al., 2003), and placenta (Fukuchi et al., 2004). Placenta-derived MSCs (PMSCs) are differentiated toward the neural lineages, such as neurons, oligodendrocytes (Portmann-Lanz et al., 2010), glial cells (Martini et al., 2013), and dopaminergic neurons (Chen et al., 2009). In addition, PMSCs show more attractive characteristics for cellular therapy and transplantation than other tissue-derived MSCs owing to their abundance, easy accessibility, fewer ethical concerns, noninvasiveness to the donors, and low immunogenicity *in vitro* and *in vivo* (Bailo et al., 2004; Yen et al., 2005; Vegh et al., 2013). Furthermore, PMSCs show an additional immunomodulatory capability over bone marrow, adipose, and umbilical MSCs (Lee et al., 2012; Talwadekar et al., 2015). Thus, PMSCs are widely applied to preclinical and clinical trials, including cardiovascular, neurological, bone and cartilage, and intestinal inflammatory diseases (Torre et al., 2019). To date, 54 clinical trials involving PMSCs are registered on ClinicalTrials.gov.

Previous works reveal that MSCs are heterogeneous under seeming homogeneity within and across different tissues at single-cell resolution (Zheng et al., 2020). The heterogeneity of MSCs may affect the therapeutic effect and give rise to inconsistencies in MSC-based clinical trials (Phinney, 2012). The use of certain functional subpopulations of MSCs may reduce the presence of interfering cells in an attempt to improve their particular ability for certain situations (Mo et al., 2016) while advanced insights into their properties and optimal selection for clinical indications require a deeper understanding of the molecular processes involved in MSCs. Therefore, there is a necessity to understand the full repertoire of MSCs and their gene expression profiles and accessible chromatin profile characteristics as the most basic and critical step to design more effective therapy strategies.

Single-cell sequencing, including single-cell RNA sequencing (scRNA-seq) and assay for transposase-accessible chromatin sequencing (scATAC-seq) are powerful tools to explore the molecular and cellular heterogeneity of MSCs derived from various tissues. It is reported that MSCs derived from umbilical cords possess limited heterogeneity during *in vitro* expansion by analysis of 361 culture-expanded MSCs and proved that cell heterogeneity is dominated by cell cycle status (Huang et al., 2019). It is reported that heterogeneity of gene expression and distinct subpopulations exist in human primary Wharton’s jelly–derived MSCs by scRNA-seq (Sun et al., 2020). Moreover, our previous study reveals molecular heterogeneity in human umbilical cord tissue and culture-expanded MSCs using scRNA-seq (Wang et al., 2021). However, unlike the bone marrow-, adipose-, and umbilical cord-derived MSCs (Liu et al., 2019; Sun et al., 2020; Wang et al., 2021), our knowledge about the heterogeneity of PMSCs at the single-cell level is still limited, especially the intersection between transcriptome and chromatin accessibility. Here, we integrate scRNA-seq and scATAC-seq tools to explore the molecular processes involved in PMSCs and provide a comprehensive and advanced resource revealing the cellular heterogeneity of PMSCs at single cell multiomics level.

## Materials and Methods

### Single-Cell Dissociation and Cell Culture

The human full-term placenta from five donors were mechanically separated before serial enzyme digestions as previously reported (Fukuchi et al., 2004). Briefly, the placenta villus tissue was washed using DPBS 2–3 times and sliced into 2 mm^3^ or smaller fragments. Then, the samples were dissociated in Dulbecco’s modified Eagle medium (DMEM, Gibco) with 100 U/ml collagenase type IV and 1% penicillin streptomycin solution (P/S). After 1 h of incubation at 37°C, 0.05% TrypLE™ Express (Thermo Fisher Scientific) was added for another 15 min incubation. The dissociation was terminated by adding 2 ml standard MSC culture medium (DMEM +10% FBS +2 mM Gln +1% P/S). Cell suspension was centrifugated at 300 g for 5 min, and the supernatant was discarded. The cell pellet was resuspended in standard MSC culture medium and cultured at 37°C in a 5% CO_2_ incubator. The cells were dissociated using TrypLE™ Express and passaged every 2–3 days five times. Cells were harvested when reaching around 80% confluence at passage 5.

### Library Preparation for scRNA-Seq and scATAC-Seq

The PMSCs at passage five were filtered through a 40 μm cell strainer and washed twice using DPBS before trypan blue staining. Then, the cell suspension with 80% viability or above was processed using 10X Genomics GemCode Single Cell Platform in 0.4% BSA–DPBS at 8 × 10^5^ cells/ml. Briefly, 10,000 cells from each sample were loaded to respective channels. The cells were then partitioned into gel beads in emulsion in the GemCode instrument, followed by reverse transcription, cDNA amplification, shearing, and adaptor-sample index attachment. Then, the 10X Genomics libraries were further prepared for the DNBSEQ platform as previously reported (Wang et al., 2021). Briefly, the single-cell libraries were amplified using 10 cycles of polymerase chain reaction (PCR) and circularized by incubating with splint oligo (BGI) and T4 DNA ligase (BGI), followed by fragment size selection with PEG32 (BGI), rolling circle amplification (RCA), and sequenced using 100 bp paired-end on the DNBSEQ platform.

The PMSCs at passage 5 (from three of a total five samples in our study) were filtered through a 40-μm cell strainer, and the cell suspension with 80% viability or above was lysed and nuclei prepared based on a previous study (Yu et al., 2021). Approximately 100,000 nuclei were mixed with 25 μl transposition reaction mixture containing 10 mM TAPS-NaOH (pH 8.5), 5 mM MgCl_2_, 10% DMF, and 4 μl of in-house Tn5 transposase. Then, they were subjected for single-cell ATAC library preparation using the DNBelab C Series Single-Cell ATAC Library Prep Set (MGI, #1000021878), which includes droplet encapsulation, preamplification, emulsion breakage, ATAC reads captured beads collection, DNA amplification, and purification. The single cell ATAC sequencing libraries were sequenced on the DNBSEQ platform at China National GeneBank (CNGB). Read structure was 70 bp for read1, inclusive of 10 bp cell barcode1, 10 bp cell barcode2, and 50 bp insert DNA; 50 bp for read2, and 10 bp for sample index.

### Flow Cytometry

The culture-expanded PMSCs at passage 5 were harvested and dissociated into single cells by 0.05% TrypLE™ Express. To determine cell surface antigen expression, the cells were processed with the Human MSC Analysis Kit (BD Stemflow™) and incubated with antibodies, including CD73, CD90, CD105, CD34, CD45, CD11b, CD19, and HLA-DR. Upon completion of the incubation, the cells were analyzed using a flow cytometer (BD Biosciences) and gated by forward and side scatter.

### Immunofluorescence Staining

PMSCs at passage 5 were fixed in 4% paraformaldehyde in PBS for 10 min and permeabilized with 0.5% Triton X-100 in PBS for 5 min at room temperature. After 120 min blocking with 3% BSA (SIGMA), cells were incubated with primary antibody overnight at 4°C. The next day, cells were washed and stained with secondary antibodies (1:300, goat antirabbit IgG-Cy3; or 1:300, goat antimouse IgG-FITC) for 60 min at room temperature and then washed three times with phosphate-buffered saline (PBS). The primary antibodies for respective cells include PRDM1 (1:100, Abcam), CXCL8 (1:300, SANTA CRUZ), TOP2A (1:200, Abcam), MKI67 (1:100, Abcam), DEDD2 (1:100, Abcam), THY1 (1:100, Abcam), CITED2 (1:100, Abcam), and IGFBP6 (1:100, Abcam). Cell nuclei were counterstained with DAPI (4′,6-diamidino-2-phenylindole) (1:500). The images were captured using Olympus IX73 and further analyzed with ImageJ software.

### Single-Cell RNA Sequencing and Data Processing

Single-cell RNA-seq FASTQ data were aligned to the GRCh38 human genome, and unique molecular identifiers (UMIs) were counted by Cell Ranger Software (Zheng et al., 2017) (cellranger-2.0.0, 10x Genomics). Genes that were expressed in less than 0.1% of total cells were removed. Cells with a detected gene number less than 800 or percentage of reads that mapped to the mitochondrial genome higher than 10% were filtered out. In addition, cells defined as outliers using a boxplot for gene number statistics were also removed. Data normalization, highly variable feature identification, dimensionality reduction, clustering, and tSNE visualization were performed with the Seurat 3.2 R package (Stuart et al., 2019). Differentially expressed genes (DEGs) of each cluster were defined by the FindAllMarkers function in Seurat with the parameter test.use = “bimod.” The aample batch effect was corrected by the IntegrateData function, cell cycle phase assignment for each cell was generated by the CellCycleScoring function, and the cell cycle batch effect was corrected by the ScaleData function with the parameter vars.to.regress = “CC.Difference” within Seurat. Constructing the trajectory and ordering single cells in pseudotime were performed with monocle (Qiu et al., 2017) (Version 2.10.1) using the top 2500 highly variable genes found by Seurat. Cluster0, one, and two were down-sampled to have the same cell number as Cluster3.

### Single-Cell ATAC Sequencing and Data Processing

The raw sequencing reads were processed by PISA^1^ and aligned to the hg38 reference genome by the BWA mem function (Li, 2013) and then deconvoluted using bap2 (Lareau et al., 2019) to create the fragment file of each scATAC-seq library for the following analysis. The TSS enrichment score and fragments of each single cell were calculated by ArchR (Granja et al., 2021) (version 0.9.5). Cells with a TSS score less than 4 or total fragments fewer than 1000 were filtered. Then, we filtered doublets based on the doublet score calculated by the function “addDoubletScores” and “filterDoublets” in ArchR. The parameter “filterRatio = 2” was used. Dimensionality reduction and clustering were also performed with ArchR using an iterative latent semantic indexing (LSI) clustering. Briefly, we created 500-bp windows tiled across the genome and determined whether each cell was accessible within each window. Next, we performed LSI dimensionality reduction with parameter “iterations = 7, dimsToUse = 1:15 sampleCells = 15,000” using the addIterativeLSI function followed by Harmony (Thi et al., 2020) for sample batch correction. Then, Seurat’s FindClusters function with parameters ‘‘reducedDims = ‘Harmony’, method = ‘Seurat’, resolution = 0.1″ was used for clustering. Gene scores were calculated by the addGeneScoreMatrix function with default parameters, and the different gene scores of each cluster were calculated by getMarkerFeatures function using ArchR. For each cluster, peak calling on the Tn5-corrected insertions (each end of the Tn5-corrected fragments) was performed using the MACS2 (Zhang et al., 2008) callpeak command with the default parameters. Then, the marker peaks of each cluster were defined by the getMarkerFeatures function, and Motif enrichment was performed by the FindMotifs function in Signac (Stuart et al., 2020) (Version 1.1) with parameter “background = NULL” in the significant marker peaks (FDR≤0.01, Log2FC≥1). TF activity was measured by chromVAR(Schep et al., 2017). Peak-to-gene links were identified by the addPeak2GeneLinks function (reducedDims = “Harmony”, dimsToUse = 1:15), Only links with FDR <0.0001, corCutOff >0.4, and varCutOff >0.25 were selected by the plotPeak2GeneHeatmap function.

### GO Enrichment Analysis

Gene ontology (GO) term analysis was performed by DAVID (Huang et al., 2009b; 2009a). GO terms with a *p*-value less than .05 were considered significantly enriched. The DEGs of each cluster in scRNA-seq and the differentially accessible genes based on gene score were used as input.

### Integration of scRNA-Seq and scATAC-Seq Data and Label Transfer

Integration of scRNA-seq and scATAC-seq was performed by the FindTransferAnchors function from the Seurat package. Briefly, it can align cells from scATAC-seq with cells from scRNA-seq by comparing the scATAC-seq gene score matrix with the scRNA-seq gene expression matrix to capture the shared feature correlation structure between two data sets.

### Transcription Factor Regulons Predicted Using SCENIC

We predicted transcription factor regulons using SCENIC (Aibar et al., 2017) following the standard pipeline as described previously. The gene expression matrix of PMSCs was used as input.

### Hierarchical Clustering Analysis

First, we selected the common genes between the gene expression data matrix and gene score matrix. We then normalized the mean gene expression/accessibility for each cluster to between zero and one using the maximum difference normalization method. Next, we performed hierarchical clustering analysis using the “hclust” function based on the Manhattan distance calculated by the “dist” function in R.

## Results

### The heterogeneity of PMSCs Revealed by scRNA-Seq

**FIGURE 1:**
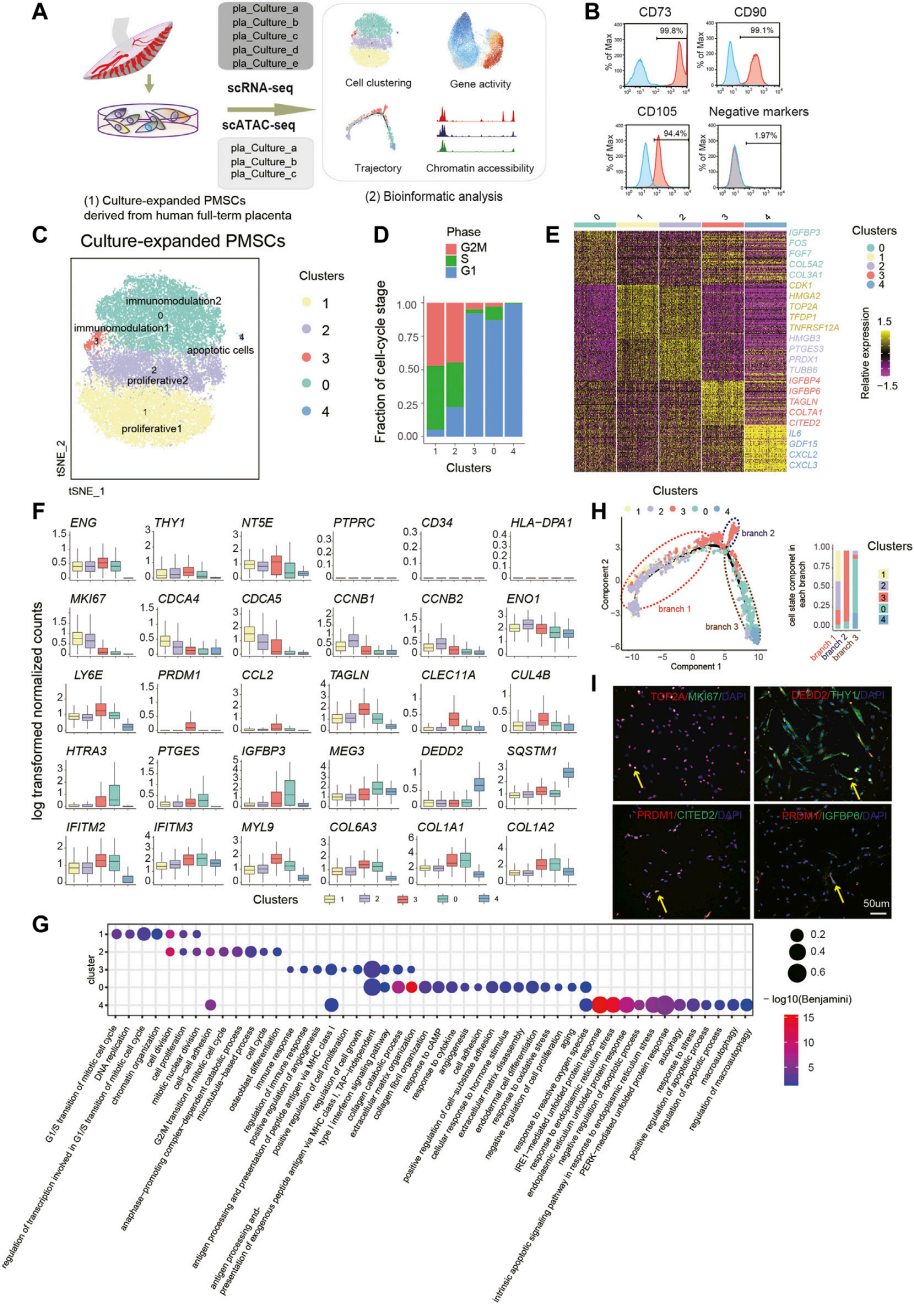
Single-Cell Transcriptome Analysis of PMSC. **(A)** Schematic overview of the workflow. MSCs derived from human placenta were processed for scRNA-seq (n = 5) and scATAC-seq (n = 3). **(B)** Representative flow cytometric histogram of PMSCs showing the presence of positive MSC markers (CD73, CD90 and CD105) and absence of negative MSC markers (CD34, CD45, CD11b, CD19, and HLA-DR were merged) **(C)** t-SNE visualization of 31,219 PMSC cells from five samples reveals heterogeneous cell states at the single-cell RNA seq level. Each dot represents a single cell (n=31,219), colored by its corresponding cluster. **(D)** Bar plot showing the fraction of cell cycle component in each cluster (bottom). **(E)** A heat map shows genes (rows) that are differentially expressed across five clusters, colored by relative gene expression (z-score). Gold: high expression; Purple: low expression. Representative genes are highlighted (p<.05, logFC>0.25, top90 in each cluster). **(F)** Boxplot showing the expression level of selected representative DEGs in five clusters. **(G)** GO terms enrichment of DEGs respective to indicated PMSC clusters. **(H)** Pseudotemporal developmental trajectory of PMSCs inferred by Monocle. Bar plot showing the fraction of each cluster component in each branch (bottom). **(I)** Immunostaining of MKI67, TOP2A, DEDD2, THY1(CD90), PRDM1, CITED2, and IGFBP6 in PMSCs.

To explore the heterogeneous states of PMSCs at single-cell resolution, culture-expanded human PMSCs (passage 5) were subjected to flow cytometry, scRNA-seq (*n* = 5, pla_Culture_a/b/c/d/e) and scATAC-seq (*n* = 3, pla_Culture_a/b/c) (Figure 1A). The flow cytometry results show high expression levels of MSC positive markers in PMSCs, including CD105 (ENG), CD73 (NT5E), and CD90 (THY1), and a lack of expression of MSC negative markers, including CD45 (PTPRC), CD34, CD11b (ITGAM), and HLA-DR (Figure 1B), which is consistent with the standard criteria for defining MSCs according to the International Society for Cellular Therapy (ISCT) in 2006 (Dominici et al., 2006). After quality control (see Methods), we kept 31,219 single cells for further transcriptomic analysis, namely, a median of 62,800 UMI counts, 6832 genes per cell, an average of 6243 cells and 209,769 mean reads per donor (Supplementary Table S1; Supplementary Figure 1B).

To generate a census of PMSCs populations, the sample batch and cell cycle effects were well excluded before deeper data mining (Figure 1D; see Methods). Five PMSC clusters with distinct features were identified based on the DEGs and the GO enrichment analysis (Figure 1C). Intriguingly, the well-known cell proliferation marker MKI67 was highly expressed in both Cluster1 and Cluster2, indicating their high proliferative capacity (Figure 1F). Besides this, genes that are strongly associated with cell proliferation and growth (e.g., *HMGA2* and *TOP2A*) (Yu et al., 2013; Lee and Berger, 2019) and cell division (e.g., *CDCA4* and *CDCA5*) were expressed at high levels in Cluster1 (Figure 1E, F). In Cluster2, we observed that *CCNB1*, *CCNB2*, and *EN O 1* were differently expressed (Figure 1E, F). A previous study shows that *CCNB1* and *CCNB2* are cell-cycle regulatory genes (Gong and Ferrell, 2010), and especially *CCNB1* is predominantly expressed in the G2/M phase of cell division (Xing et al., 2021); moreover, *CCNB1* is a cell–cell adherence term associated gene along with *EN O 1.* In line with the gene expression results above, GO analysis of the DEGs in these two clusters showed several overlapped GO terms, including cell division, cell proliferation, and mitotic nuclear division (Figure 1G). However, Cluster1 DEGs were enriched in the G1/S transition of the mitotic cell cycle and Cluster2 DEGs in the G2/M transition of cell cycle, anaphase of mitosis related terms. Notably, two MSC-featured terms, cell–cell adhesion and osteoblast differentiation potency, were only observed in Cluster2 (Figure 1G). These results suggest that Cluster1 and Cluster2 might be highly proliferative subpopulations. Cluster1 cells could be in a highly proliferative, multipotent progenitor cell state with 94.8% of cells in the G2M/S phase (Figure 1C), and Cluster2 might be in a precommitted MSC cell state, which are poised to differentiated cell state with 77.8% of cells in G2M/S phase (Figure 1C). Above all, we named Cluster1 and Cluster2 as proliferative1 and proliferative2, respectively.

Besides this, we identified two immunomodulatory-related PMSCs subgroups, Cluster3 and Cluster0, which were named as immunomodulation1 and immunomodulation2, respectively. Several well-known immunomodulation-associated genes (e.g., IFITM2, IFITM3, and MYL9) and collagen genes (e.g., COL6A3, COL1A1, and COL1A2) were commonly expressed in both clusters (Figure 1F). Moreover, immunomodulation1 and immunomodulation2 cells showed their own specific immunomodulation signatures. For example, CCL2, PRDM1, and LY6E were specifically highly expressed in immunomodulation1, and HTRA3 and PTGES were solely expressed in immunomodulation2 (Figure 1F). All these genes are involved in different immunomodulating processes based on previous studies (Rafei et al., 2008; Fu et al., 2017; Wang T. et al., 2020; Ji et al., 2020; Pfaender et al., 2020) (Figure 1E). In line with this, we also observed overlapped features of immunomodulation and extracellular matrix-related GO terms in the two clusters, which are important for the maintenance of MSC functions (Figure 1G). Besides this, a few more immunomodulation-related terms were enriched in immunomodulation1, whereas immunomodulation2 showed more significant features in extracellular matrix organization–, collagen formation–, and cell adhesion–related terms (Figure 1G).

In addition, immunomodulation2 showed high expression of *FOS*, *IGFBP3*, and *MEG3* (Figure 1E, F), which are reported to be involved in promoting MSC differentiation (Komori, 2006; ZHUANG et al., 2015; Deng et al., 2018). In contrast, immunomodulation1 showed high expression of *CLEC11A*, *TAGLN*, *CUL4B*, *IGFBP6*, and COX7A1 (Figure 1E, F). *CLEC11A*, also known as stem cell growth factor, promotes the proliferation and differentiation of hematopoietic stem/progenitor cells (Wang M. et al., 2020). Moreover, studies indicate that *CLEC11A* can promote the proliferation of islet cells and as the potently pro-osteogenic gene to mark the related adipogenic population (Merrick et al., 2019; Shi et al., 2019). *COX7A1*, as a potential mammalian embryonic-fetal transition (EFT) marker that is upregulated in post-EFT murine and adult stem cells (West et al., 2018), was also highly expressed in immunomodulation1. Previous studies show that the addition of *IGFBP6* significantly increases pluripotency and differentiation-associated markers in PMSCs, and silencing *IGFBP6* decreases both of them (Aboalola and Han, 2017). In addition, knocking down *IGFBP6* in vascular smooth muscle cells significantly reduces cell proliferation and induces S phase arrest in the cell cycle (Wang Z. et al., 2020)*.* In line with this, immunomodulation2-specific genes were more enriched in the negative regulation of cell proliferation, aging, response to reactive oxygen species et al., whereas immunomodulation1 was more related to positive regulation of cell proliferation and regulation of cell growth. All the above results suggest that immunomodulation1 could be related to the immunomodulation capacity cell state that shares many properties with immunomodulation2 but is more energetic, and immunomodulation2 could be in a mature immunomodulation capacity cell state with more committed differentiation.

Meanwhile, we also observed a small group of cells, Cluster4, contributing 0.33% of the total PMSCs cells but with high expression of apoptotic-related genes, including *GDF15*, *SQSTM1*, and *DEDD2*. Besides this, *CXCL2*, *CXCL3*, and *IL-6/24* were also highly expressed in this cluster (Figure 1E, F). The GO enrichment analysis showed that DEGs upregulated in Cluster4 were highly enriched in autophagy and apoptosis-related terms (Figure 1G), which was consistent with apoptotic MSCs showing immunomodulation functions *in vivo* (Galleu et al., 2017). Thus, we inferred that Cluster4 might be an apoptotic cell state.

We observed distinct subpopulations with specific molecular features, and a previous study shosn that *in vitro* expansion of MSCs induces spontaneous differentiation with expression of developmental markers and tissue-specific genes (Tsai et al., 2011), which inspired us to dissect the trajectory of the above clusters. Thus, we performed the pseudotime analysis using the PMSCs subpopulations. Consistently, our unsupervised trajectory analysis by Monocle2 showed that proliferative1 and proliferative2 were at the root of the trajectory. Immunomodulation1 was located in the middle of the trajectory with a minor branch, and immunomodulation2 was clustered in the further branches with apoptotic cells at the end (Figure 1H). The above results, combined with immunostaining of MKI67, TOP2A, PRDM1, CITED2, IGFBP6, DEDD2, and THY1 (Figure 1I), clearly showed the heterogeneous cellular states of PMSCs at the single-cell transcriptional level.

### Single-Cell Chromatin Accessibility Landscape of PMSCs

**FIGURE 2:**
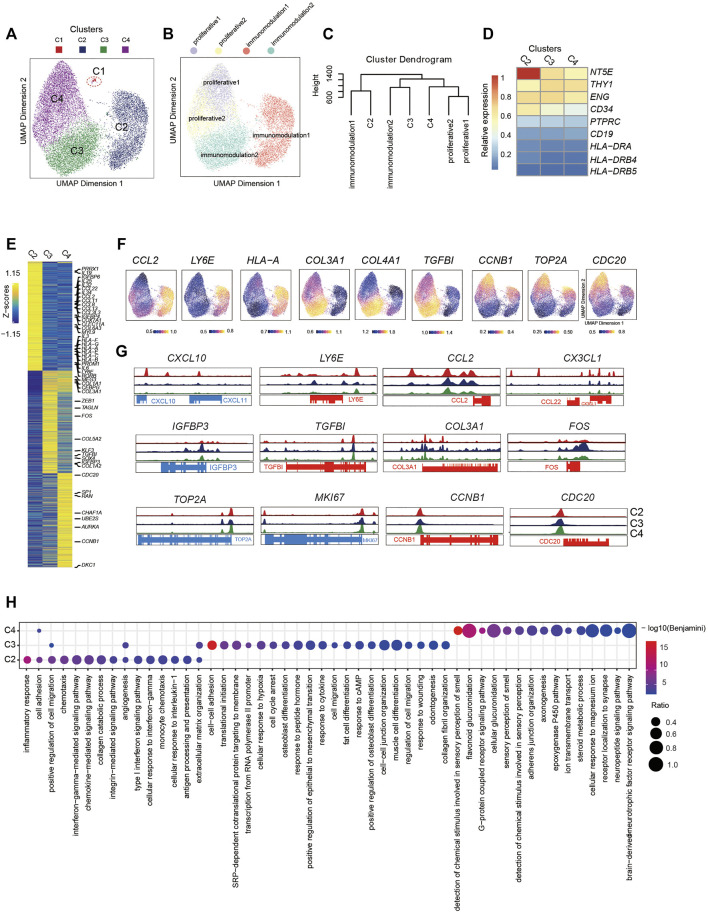
Single-Cell Chromatin Accessibility Analysis of PMSCs **(A)** UMAP visualization of 17,410 PMSC cells from three samples reveals heterogeneous cell states at the single-cell ATAC seq level. Each dot represents a single cell, colored by its corresponding cluster. **(B)** The same UMAP visualization shown in **(A),** but each cell is colored by the predicted corresponding RNA cell states (n=17,356). C1 was removed. (Right) **(C)** Dendrogram showing relationships among subclusters from scATAC-seq and scRNA-seq. The variable features of scRNA-seq data identify by Seurat presenting in GeneScoreMatrix and normalized RNA expression matrix were used. The mean score of each gene in each cluster were scaled. **(D)** Chromatin accessibility for the positive MSC markers (CD73, CD90, and CD105) and negative MSC markers (CD34, PTPRC(CD45), CD19, and HLA-DR). **(E)** scATAC-seq heat map of differentially activity gene across five clusters, colored by relative gene-activity scores (z-score). Gene-activity was converted from accessible peaks calculated in ArchR using Cicero. Gold: high activity; blue: low activity. Representative genes from scRNA-seq and relative function are highlighted. (log2 fold change (LFC) > 0.15 and false discovery rate (FDR)<0.01) **(F)** UMAP visualization show the gene activity of representative genes select from scRNA-seq in corresponding scATAC-seq clusters. **(G)** Aggregated scATAC-seq tracks denoting marker chromatin accessibility peaks for each cluster. **(H)** GO terms enrichment of different activities gene respective to indicated scATAC-seq clusters.

To explore the epigenetic characteristics of PMSCs, we harvested 17,410 scATAC-seq data sets from three individuals (pla_Culture_a/b/c) after quality control (Figure 1A; see Methods). The integrated single-cell ATAC-seq data set from all three samples showed high numbers of unique nuclear fragments per cell and signal-to-background ratio indicated by transcription start site (TSS) score (Supplementary Figure 1D). In addition, the distribution of expected fragment size with strong enrichment signal at TSS (Supplementary Figure 1E) indicated the high data quality used in our study. We identified four distinct clusters (C1, C2, C3, C4) (Figure 2A) when the data were projected to a low-dimensional space using ArchR based on the characteristics of peaks after batch effect removal by Harmony (Supplementary Figure 1F). C1 was fewer than 100 cells (54 cells, 0.3% of total) and showed no unique molecular feature pattern. Thus, only the C2, C3, and C4 were used for the following analysis.

To further dissect the epigenetic characteristics of PMSC subgroups and review the association between the transcriptional and epigenetic layer, we transferred cell states from scRNA-seq data to scATAC-seq data based on gene expression and the gene body accessibility matrix (see Methods; Figure 2B). Two proliferative clusters, proliferative1 and proliferative2 in scRNA-seq data, were both transferred to one scATAC-seq cluster, C4, although they showed distinct cell states at the transcriptional level. The small immunomodulation1 cluster (1.38% in scRNA-seq data, Figure 1C) was transferred to a large cluster, C2, in scATAC-seq data (5,031 cells, 29% of total); moreover, immunomodulation2 was assigned into C3.

To further identify the assignment relationship of the cell states, we performed hierarchical clustering analysis for both scRNA-seq and scATAC-seq subgroups based on the distance using all common genes (see Methods). The hierarchical clustering results also showed that proliferative1 and proliferative2 were classified together with C4, immunomodulation1 and immunomodulation2 were gathered with C2 and C3, respectively (Figure 2C). These data further confirm the predicted annotations by Seurat. The above results suggest that the heterogeneity of cell states in PMSCs could also be reflected by chromatin accessibility, and there are corresponding correlations but significant differences across transcriptional and epigenetic levels.

To further explore the relationship between gene expression and chromatin accessibility of PMSCs subgroups, we identified genes with differential activity based on the inferred gene-activity score (Figure 2E) and performed GO enrichment analysis (Figure 2H). We observed that the enriched genes in immunomodulation1 population, including *PRRX1*, *PRDM1*, *CCL2*, *LY6E*, *IGFBP4*, *IGFBP6*, *COX7A1*, and *CLEC11A*, showed higher accessibility in C2 than others (Figure 2E–G). Moreover, some cytokine superfamily members, including the CCL chemokine family, CXC chemokine family, interleukins family, and HLA major histocompatibility complex (MHC) class I protein members, only had significantly differential chromatin accessibility in C2. Please note that not all of these genes were consistently highly expressed at the transcriptional level, suggesting that the chromatin accessibility of immunomodulatory-related genes may be poised and keep more open accessibility for initiating the expression of these genes in PMCSs (Figure 2E). Note that immunomodulation-related terms, including “type I interferon signaling pathway,” “monocyte chemotaxis,” “antigen processing and presentation,” and “interferon−gamma−mediated signaling pathway,” were only observed in C2 (Figure 2H).

In addition, MSC differentiation and extracellular matrix–related genes that were highly enriched in immunomodulation2 in the scRNA-seq data (e.g., *FOS*, *SOX4*, *MEG3*, *IGFBP3*, *COL3A1*, and *COL5A2*), showed higher accessibility in C3, indicating the differentiation but not immunomodulation characteristics of PMSCs were more prominent in C3 at the chromatin accessibility level (Figure 2E–G). It is worth mentioning that, GO terms including “cell cycle arrest,” “cAMP response,” and “cytokine response,” were also enriched in C3 as what we observed in immunomodulation2 with scRNA-seq data. Besides this, functional characteristics of classic MSCs, such as “extracellular matrix organization,” “cell–cell adhesion,” and “cell migration,” MSC differentiation potential–related terms were relatively upregulated in C3. These results suggest that immunomodulation-related genes showed a clearer chromatin accessibility signature than those at the transcriptional level in PMSCs, and the chromatin accessibility of immunomodulation-related genes is being poised for immunomodulation specification.

**FIGURE 3:**
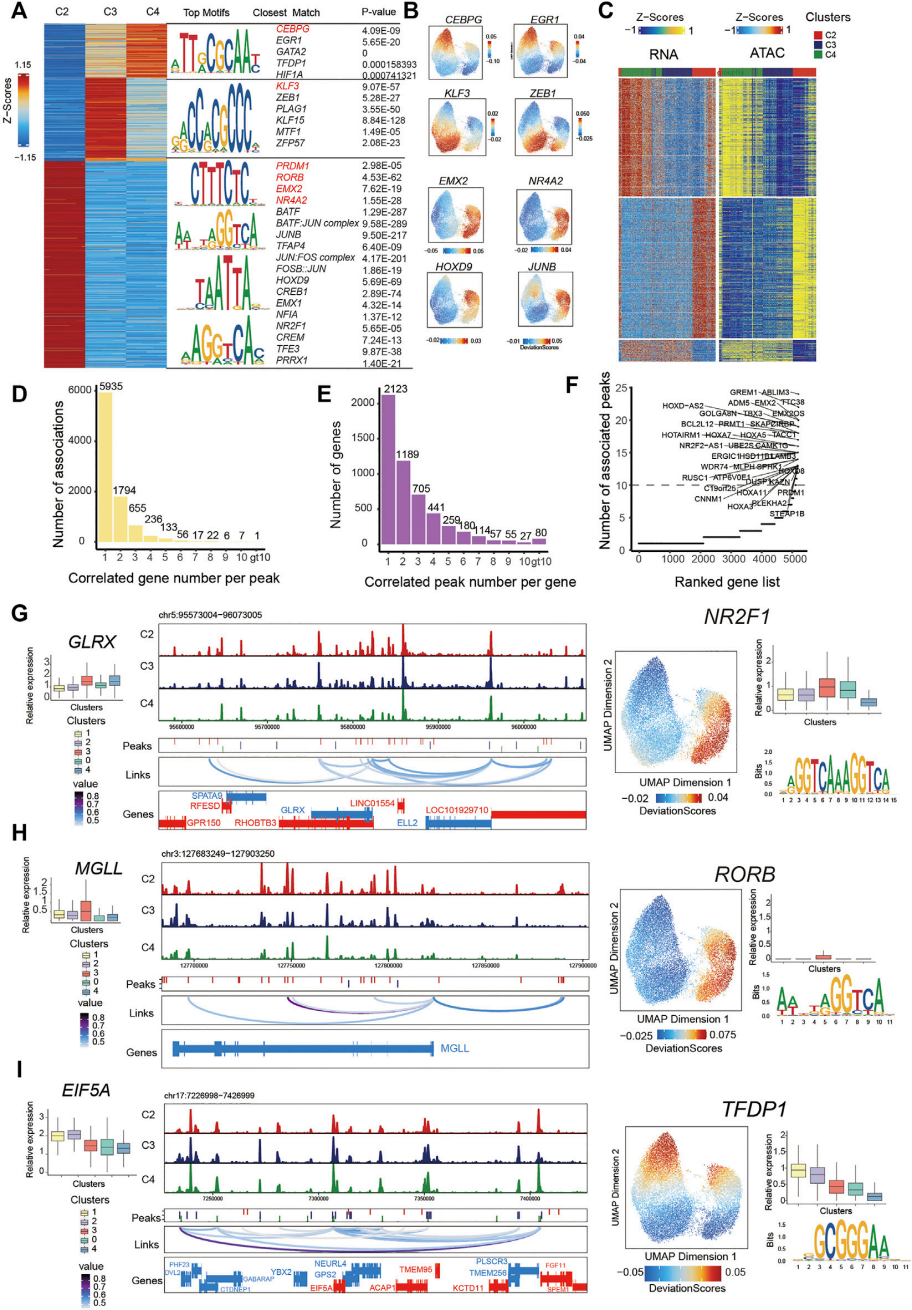
Integrated analysis of cell state–specific epigenetic regulators in inferred PMSC subgroups.**(A)** Heat map representing PMSC cell states marker peaks. Each row represents an individual marker peak, colored by the normalized marker peak accessibility score (Z-score) (Left). Transcription factor motifs and transcription factor and P-value enriched in each cell state marker peak sequence. Transcription factor motifs selected in red. **(B)** The same UMAP visualization shown in Figure 2A, but each cell is colored by the enrichment of TF activity score (deviations) calculated in ArchR using ChromVAR. **(C)** Heat maps of 13,863 significant peak-to-gene links across cell states (FDR<0.0001; corCutOff > 0.4; varCutOff > 025 when selecting links by plotPeak2GeneHeatmap function). Top, peak-to-gene links that are identified almost within C4. Middle, peak-to-gene links that are unique to C2. Bottom, peak-to-gene links identified in both C3 and C4. **(D)** The number of significant peak–gene links for all peaks. **(E)** The number of significant peak–gene links for all genes. **(F)** The number of significantly correlated peaks for each gene. Putative DORCs are highlighted. **(G-I)** Aggregated scATAC-seq tracks showing genomic regions near GLRX(G), MGLL **(H)**, and EIF5A **(I)** genes. Different peaks in clusters are shown in the second line. The loop in the third line height represents the significance of peak-to-gene links (corCutOff = 0.45, FDRCutOff = 1e-04, varCutOffATAC = 0.25, varCutOffRNA = 0.25). The RNA expressions are present on the left boxplot. The motif enrichment for associated peaks (shown in **(C)**) are shown in the right and the UMAP show the enrichment of TF activity score (deviations); the boxplot shows the RNA expression of the enriched TF.

As expected, cell proliferation– and cell cycle–related genes, such as *TOP2A*, *MKI67*, *CCNB1*, *CDC20*, A*DKC1*, *AURKA*, and *UBE2S*, showed differential chromatin accessibility in C4, which confirmed the definition of subpopulations in scATAC-seq data based on the transfer method as described above (Figure 2E–G). However, the cell cycle–related genes did not show as significant a difference at the chromatin accessibility level among the three clusters as those at the transcriptional level (Figure 3G). Interestingly, genes with a highly predicted gene score in C4 were significantly enriched in sensory-, neuro-, and cell adhesion–related GO terms with few terms related to the cell cycle (Figure 2H). Above all, we inferred that cell cycle gene-related heterogeneity has more distinct characteristics at the transcriptional level than those at the chromatin accessibility level in PMSCs.

Collectively, the above results suggest that transcriptomic and epigenetic data could largely reflect each other in cell state identification. However, these two omics layers are not always consistent and are able to provide complementary information for better understanding of the heterogeneity of PMSCs.

### Characterization of the Cell State–specific Epigenetic Regulators in Inferred PMSCs Subgroups

To characterize the cell state–specific regulatory networks involved in regulating chromatin accessibility across different cell states, we conducted a Wilcoxon test to find differential peaks for each PMSC subpopulation. In total, we found 55,149 significantly accessible chromatin peaks (Log2FC > 1 and FDR <0.05) in at least one PMSC subpopulation across three cell states (clusters C2, C3, and C4) (Figure 3A). These peaks were clustered into three major groups representing the specific chromatin-accessible sites of each PMSC subgroup. Based on the marker peaks in each subgroup, we then performed motif enrichment using the FindMotifs function in Signac. As a complementary approach, some of them also showed high TFs activity calculated by ChromVAR at a per-cell level. Using this combined motif enrichment approach, we observed enrichment of the binding motif of *PRDM1*, *RORB*, *EMX2*, *BATF:JUN* complex, *JUNB*, and *JUN:FOS* complex (AP-1 family members) in C2; *KLF3*, *PLAG1*, and *ZEB1* in C3; and *CEBPG*, *EGR*1, *GATA*2, and *TFDP1* in C4, respectively (Figure 3A,B).

The pattern of chromatin accessibility reflects the possible physical interactions among enhancers, promoters, insulators, and chromatin-binding factors, all of which could cooperatively regulate gene expression (Klemm et al., 2019). Cell type–specific gene expression in eukaryotic cells was regulated by *cis*-acting DNA elements, including enhancers and promoters, and *trans*-acting factors, such as transcription factors (Roadmap Epigenomics Consortium et al., 2015). To infer the *cis*-regulatory elements of target genes in chromatin accessibility peaks, we first identified the putative 499,568 peak-gene linkages that significantly paired (within 250 kb of a gene promoter, FDR <0.05) and used the most significant 13,863 peak–gene association for the following analysis (correlation >0.45; FDR < 1e-04; the minimum variance quantile of the ATAC peak accessibility and the minimum variance quantile of the RNA gene expression >0.25; Figure 3C). These results reveal specific peak-to-gene links in C2 and C4 and a conserved subset that is shared across both C3 and C4 (Figure 3C). Meanwhile, we found that most peaks were only associated with one gene, and most genes were only associated with one peak (Figure 3D,E), which is in line with a previous study (Ma et al., 2020).

A previous study demonstrates that domains of regulatory chromatin (DORCs, with an exceptionally large (>10) number of significant peak-gene associations) referring to peak–gene–associated regions with high density, were strongly enriched for lineage-determining genes (Ma et al., 2020). To find out the key cell state–determining genes, we inferred DORC-regulated genes as previously reported (Ma et al., 2020) (Figure 3F). As expected, a subset of genes was significantly associated with more than 10 peaks. For example, *GREM1*, *MGLL*, *GLRX*, *PRMT1*, *EIF5A*, and *PRDM1* showed both a differential expression pattern and chromatin accessibility state across different cell subgroups. A total of 22 peaks were significantly associated with *GREM1* and 11 peaks with *MGLL* and *GLRX*. These genes all showed differential chromatin accessibility and gene expression in immunomodulatory-capability cell subgroups. These results are consistent with the previous report that *MGLL* and *GLRX* are associated with immunity in cancer (Xiang et al., 2018; Chang et al., 2020) and *GREM1* inhibits osteogenic differentiation, senescence, and BMP transcription of adipose-derived stem cells (Liu et al., 2021). Additionally, 18 peaks were significantly associated with *PRMT1* and 11 peaks with *EIF5A*, and they both showed differential chromatin accessibility and gene expression in proliferative subgroups (Figure 3G–I). Consistently, previous reports indicate that *EIF5A* and *PRMT1* promote cell proliferation in the case of several human cancers (Kaiser, 2012; Song et al., 2020). Based on the above findings, we propose that these identified peaks from significant peak–gene linkages might be specific *cis*-regulatory elements for cell state–related genes.

To further dissect the *cis*-regulatory elements directing the expression of those genes, we performed motif enrichment analysis on the significantly associated peaks. We found transcription factor binding sites (TFBSs) for *NR2F1* in *GLRX-*associated peaks, *RORB* in *MGLL*-associated peaks, and *TFDP1* for *EIF5A*-associated peaks. They had high TF activity in C2 or C4 (Figure 3G–I). Based on the temporal specificity of scATAC-seq peaks and the existence of TFs motifs in these regions, we propose that those elements might be cell state–specific *cis*-regulatory elements to regulate the expression of PMSC regulators that cause heterogeneity. Overall, our data not only present some well-known TFs that are previously reported for MSC functional phonotype, but also highlight some putative TFs for regulating PMSCs heterogeneity at the epigenomic level, which may pave the way for deeper understanding of the MSC functional mechanism.

### *PRDM1* Plays a Crucial Role in Maintaining Immunomodulatory Capability by Activating *PRDM1*-Regulon Loop

**FIGURE 4:**
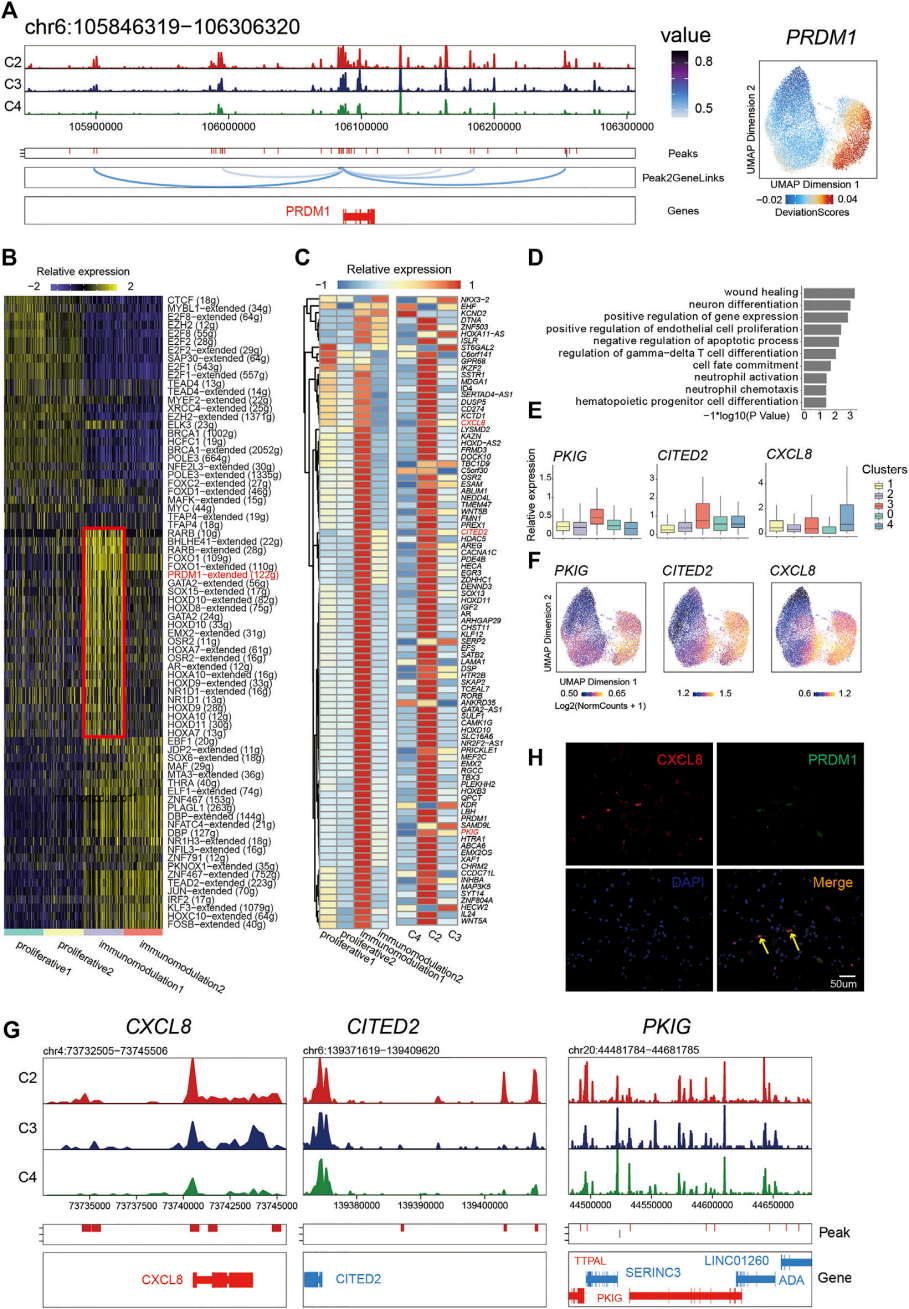
*PRDM1* played a crucial role in maintaining immunomodulatory capability of PMSC subgroup. **(A)** Aggregated scATAC-seq tracks showing genomic regions near *PRDM1* (Left). The UMAP shows the enrichment of *PRDM1* activity score (deviations). **(B)** Heat map of different regulon identified by SCENIC. **(C)** Heat map of PRDM1 target genes expression (left) and chromatin accessibility (right) **(D)** GO enrichment for *PRDM1* target genes (E) Boxplot showing the expression level of selected representative PRDM1 target genes PKIG, CITED2, and CXCL8. **(F)** The UMAP show the enrichment of PRDM1 target genes (*PKIG, CITED2*, and *CXCL8*) activity score (deviations). **(G)** Aggregated scATAC-seq tracks showing genomic regions near PKIG, CITED2, and CXCL8, respectively. **(H)** Immunostaining of PRDM1 and CXCL8 in PMSCs.

TFs play important roles in dictating the identity and fate of individual cells in multicellular organisms by differentially regulating the gene expression upon internal and external stimuli (Lee and Young, 2013). Previous studies show that *PRDM1* play vital roles in regulating the cell development and differentiation process (Turner et al., 1994; Ohinata et al., 2005; Kallies et al., 2006); however, its role in MSCs is still largely unknown. Here, we found *PRDM1* also showed up in the list of DORC-regulated genes with differential expression in the immunomodulation1 cell state and chromatin accessibility in C2 (Figure 3F; Figure 2F; Figure 4A). In addition, *PRDM1* motifs were highly enriched in differential peaks of C2 and showed high TF activity (Figure 3A; Figure 4A). Meanwhile, *PRDM1* also showed a differentially high TF regulon activity based on Single-Cell regulatory Network Inference and Clustering (SCENIC) (Aibar et al., 2017) calculated AUC scores in immunomodulation1 (Figure 4B). These results indicate that *PRDM1* might contribute to the regulation of immunomodulation cell state in PMSCs.

Thus, we further extracted the putative target genes of *PRDM1* inferred by SCENIC and identified the chromatin accessibility and expression profiles of them to investigate the relationship between the chromatin structure remodeling and gene expression in the *PRDM1* regulon network. Notably, we found that most target genes showed an enriched expression in immunomodulation1 and a significant pattern of chromatin accessibility in C2 (Figure 4C). Moreover, we observed synchronous dynamics of those targets; for instance, the target *CXCL8*, *CITED2*, and *PKIG* showed high gene expression (Figure 4E) and chromatin accessibility (Figures 4F,G), suggesting an important role in maintaining the immunomodulation cellular phenotype. In addition, the GO enrichment analysis for *PRDM1*-target genes showed that “wound healing,” “neuron differentiation,” “positive regulation of endothelial cell proliferation,” “regulation of gamma-delta T cell differentiation,” “cell fate commitment,” “neutrophil activation,” and “hematopoietic progenitor cell differentiation and neutrophil chemotaxis” were highly enriched (Figure 4D). The immunostaining result was in line with the above analysis; namely, *PRDM1* and target gene *CXCL8* were co-expressed in some PMSC single cells (Figure 4H). Collectively, our results indicate that, as a significant character in this cell state, *PRDM1*-regulon genes might be important for maintaining of immunomodulatory potential cell state in PMSCs.

## Discussion

MSCs are a promising cell source for clinical application. More and more studies indicate that MSCs, even though all meet ISCT criteria, are cell mixtures in aspects of phenotypical, functional, and biochemical characteristics. MSC subpopulations with distinctive surface markers display different biological potential and corresponding therapeutic effects (Tormin et al., 2009; Phinney, 2012; Mo et al., 2016). Even single-cell-derived colonies of human MSCs are heterogenous in morphology, self-renewing ability, and the potential for multilineage differentiation, migration, and tissue engraftment (Colter et al., 2001; Prockop et al., 2001; Ryang et al., 2006). The knowledge regarding what leads to the heterogeneity of MSCs is largely unexplored. In our study, we generated a transcriptional map and a complementary chromatin-accessibility map of human PMSCs. In line with previous multiomics studies (Ma et al., 2020), the cell state heterogeneity in PMSCs can be reflected across the transcriptional and epigenetic landscape. A previous study proposes that the mesengenic process represents a complex sequence of events (Caplan, 2008). It is reported that *in vitro* expansion of MSCs also induced spontaneous differentiation with expression of developmental markers and tissue-specific genes (Tsai et al., 2011). Consistently, we also observed such phenomenon using unsupervised trajectory analysis by Monocle2 (Figure 1H), which may be attributed to intrinsic subsets with specific molecular features existing in cultured PMSCs as well as adopting FBS in PMSCs culture medium in the current study.

Based on multiomics analysis, we reveal that PMSCs show significantly enriched immunomodulatory capability at single cell resolution. The immunomodulatory capability of PMSCs are also reported in a previous study by using traditional tools. For example, PMSCs could inhibit the inflammatory response by regulating CD4^+^ T cell and macrophage polarization, inhibiting the inflammatory factors *IFN-γ* and *IL-17*, and upregulating the anti-inflammatory factor *TGF-β* and *IL-10* expression to attenuate renal fibrosis in rats (Zhu et al., 2020).

We identified one PMSC subgroup, C2, as main immunomodulatory potential cell state at the single-cell chromatin accessibility level, in which *LY6E*, *CCL2*, *GREM1*, *PRDM1*, and many other cytokine genes showed significant chromatin accessibilities. These genes were also highly expressed in the corresponding immunomodulatory1 cell state at the single-cell transcriptional level. Furthermore, estimates of immunomodulatory-related gene activity on the basis of correlated variation in promoter and distal-peak accessibility (Cicero) broadly repeats this pattern, including *IGFBP4, IGFBP6*, *CCL2*, *PRDM1*, and *LY6E* as well as other chemokine, CXC chemokine, and interleukin family members. GO enrichment analysis for genes with differential expression from the scRNA-seq data set and differential gene activity from the scATAC-seq data set in this immunomodulatory-potential cell state indicate common terms in immune- and collagen-related biological processes (Figure 1G, Figure 2E). Collectively, immunomodulatory potential cell state characteristics in PMSCs were consistently reflected across both the transcriptional and epigenetic maps (Figure 1F; Figure 2E,F).

It is reported that the therapeutic effect of MSCs are not primarily influenced by their differentiation potential but rather by the secretion of growth factors and cytokines in many cases (Caplan, 2008). MSCs can secrete cytokines and other factors, such as *TGFβ*, *IL-6*, *CCL2*, and *HLA-G* to exert the immunomodulatory effect (Choi et al., 2014; Maffioli et al., 2017). Owing to the immunomodulatory abilities, MSCs are used for many preclinical studies and clinical trials, including graft-vs-host disease, autoimmune diseases, inflammatory illnesses, lung injuries, etc. (Ringdén et al., 2006; Li and Flavell, 2008; Lee et al., 2009; Matthay et al., 2010). It is worth noting that MSCs show their efficacy in alleviating comorbidities associated with COVID-19 by directly mitigating inflammation, reversing lung dysfunction via normalizing the pulmonary microenvironment, preventing pulmonary fibrosis, and so on (Song et al., 2021).

Besides the cytokines and chemokines mentioned above, *LY6E* and *IFITM2/3* (interferon-induced transmembrane proteins 2/3) were identified in our data with differential expression levels across the clusters (Figure 1F). It is reported that *LY6E* could control CoV infection and pathogenesis and confer immune control of viral diseases, including SARS-CoV-2 (Pfaender et al., 2020; Zhao et al., 2020). *IFITM2/3* are restriction factors that block the entry of many viruses, including SARS-CoV-2 (Lee et al., 2013; Shi et al., 2021). In addition, it is reported that MSCs have an antimicrobial role and therapeutic effects on bacterial infection–caused lung injury (Lee et al., 2013). Based on the results presented in our study, PMSCs might be a better choice with promising potential to be used in COVID-19 treatment. However, more work still needs to be done for further validation.

Interestingly, the immunomodulatory-potential cell state in the scATAC-seq data, compared with that from the scRNA-seq data, has more obvious immunomodulatory characteristics with an increased ratio in cell composition (1.38% up to 29%; Figure 1; Figure 2A) and high chromatin accessibility of immunomodulatory-related genes (Figure 2E–G). A previous study reveals that immunomodulatory activity of MSCs was seriously influenced by the inflammation microenvironment during tissue regeneration (Shi et al., 2018). Instead of being immunosuppressive in nature, MSCs might have different immunoregulatory properties depending on the immune scene and disease condition (Song et al., 2021). The fate of the implanted MSCs is locally regulated by the new environment, and their further development is selective and not directive (Pittenger et al., 2019). Prior studies observe that changes in histone modifications and chromatin accessibility for sequence-specific transcription factors might precede and prefigure changes in gene expression, and chromatin accessibility lineage-priming states could predict cell fate decisions (Rada-Iglesias et al., 2011; Lara-Astiaso et al., 2014; Ma et al., 2020). Based on our results and previous reports, we suggest that MSCs’ immunomodulatory potential might be characterized to be latent at the epigenomic level when there is no specific inflammatory stimulus. The chromatin accessibility of the immunomodulatory-related genes precedes and foreshadows gene expression by creating primed chromatin states to activate their expression and fulfill the immunomodulatory potential.

Moreover, we also present two proliferative cell states in PMSCs. Compared with immunomodulatory cell states, the proliferative cell states show increased expression of genes related to the cell cycle, cell proliferation, and mitotic nuclear division and reduced expression of differentiation-related genes. After removing the cell cycle effect, we still observed that cell cycle composition was considerably inconsistent among various groups. The cell cycle state may have intrinsic characteristics of cultured cells, and this phenomenon is also previously described in other scRNA-seq studies (Kowalczyk et al., 2015; Harman et al., 2020). Meanwhile, two proliferative cell states in single-cell transcriptome data were both transferred to C4 in the single-cell chromatin accessibility data. Moreover, cell cycle–related genes, including DKC1, AURKA, CCNB1, CDC20, and UBE2S, showed moderate but significant differential chromatin accessibility. Furthermore, other than regulation of the fibroblast growth factor receptor signaling pathway and negative regulation of cell differentiation– and cell adhesion–related terms, the extensive chromatin priming of genes was also related to nervous system development and the neuropeptide signaling pathway. It is reported that PMSCs are capable of being induced to several neural cell types (Chen et al., 2009; Portmann-Lanz et al., 2010; Martini et al., 2013) and can be used in neurological disease treatment (Torre et al., 2019). For example, PMSCs could be differentiated into neural progenitors *in vitro*, and these progenitors could further differentiate into dopaminergic neurons to alleviate asymmetric rotational behavior after being transplanted into the striatum of Parkinson’s disease model rats (Park et al., 2012). Moreover, it is demonstrated that PMSCs could modulate the inflammatory response in an Alzheimer’s disease mouse model and increase the levels of β-amyloid degrading enzymes, resulting in an improvement of memory function (Kim et al., 2013). The current accuracy of computational approaches that pair data from scATAC-seq and scRNA-seq from separately measured cells is variable (74.9% in skin and 36.7% in mouse brain) (Ma et al., 2020). A more scalable and better integrated approach, namely, not only sequencing technology but computational pairing approaches, would be useful for better understanding the relationship between the transcriptome and epigenome.

Chromatin accessibility regulates gene expression by modulating the interactions of transcriptional factors with their target DNA, which plays an essential role in establishing and maintaining cell identity (Klemm et al., 2019). Interactions among chromatin regulators, transcription factors, and *cis*-regulatory elements are the main drivers to shape context-specific chromatin accessibility and maintain the gene expression profile (Duren et al., 2017). In our work, we discovered that there were specific correlations between gene expression and peak accessibility at different cell states (Figure 3C). We linked differentially accessible regions, which were inferred as the specific *cis*-regulatory elements for cell state–related genes to DORC-regulated genes, such as *MELL*, *GLRX*, and *EIF5A.* Meanwhile, we identified the key TFs that regulate these genes using ChromVAR (Figure 3G–I). We found that *PRDM1* motifs were highly enriched in C2, and as a DORC, *PRDM1* showed significant chromatin accessibility. In addition, *PRDM1* was significantly upregulated in the immunomodulatory cell state. Moreover, target genes of *PRDM1* inferred by SCENIC had a coordinated pattern in gene expression and chromatin accessibility in the immunomodulatory cell state. The results of GO enrichment analysis showed that *PRDM1*-target genes were significantly enriched in wound healing, regulation of gamma-delta T cell differentiation, cell fate commitment, and neutrophil activation, etc. Previous studies shown that *PRDM1* serves as a master regulator of the development and differentiation of immunoglobulin-secreting B cells (Turner et al., 1994). Besides this, *PRDM1* is also important for thymocyte survival, T cell homeostasis (Kallies et al., 2006; Martins et al., 2006), T helper differentiation (Kallies et al., 2006), and cytokine production (Heinemann et al., 2014). It is also reported that *PRDM1* plays a critical role in the process of mouse germ cell lineage formation (Ohinata et al., 2005). Taken together, *PRDM1* would play a potential role in maintaining immunomodulatory capability by activating the *PRDM1*-regulon loop. Our work reveals potential regulatory factors and an important pathway for PMSC cell state commitment at the single cell multi-omics level for the first time, which may further support the application of PMSCs in regenerative and immunomodulation treatment. Future validation studies in chromatin accessibility and gene expression on PMSCs will increase our knowledge of the regulatory network associated with the heterogeneity of PMSCs and optimize the clinical application of MSCs.

## Data Availability

The data that support the findings of this study have been deposited into CNGB Sequence Archive (CNSA) (Guo et al., 2020) of China National GeneBankDataBase (CNGBdb) (Chen et al., 2020) (link: https://db.cngb.org/search/project/CNP0001689/), accession number CNP0001689.

## Footnotes

1
https://github.com/shiquan/PISA
