# Supplementary figures and images for: Integrative Single-Cell RNA-Seq and ATAC-Seq Analysis of Mesenchymal Stem/Stromal Cells Derived from Human Placenta
Source: Front Cell Dev Biol. 2022 Apr 5;10:836887. doi: 10.3389/fcell.2022.836887 (PMC9017713; doi:10.3389/fcell.2022.836887)

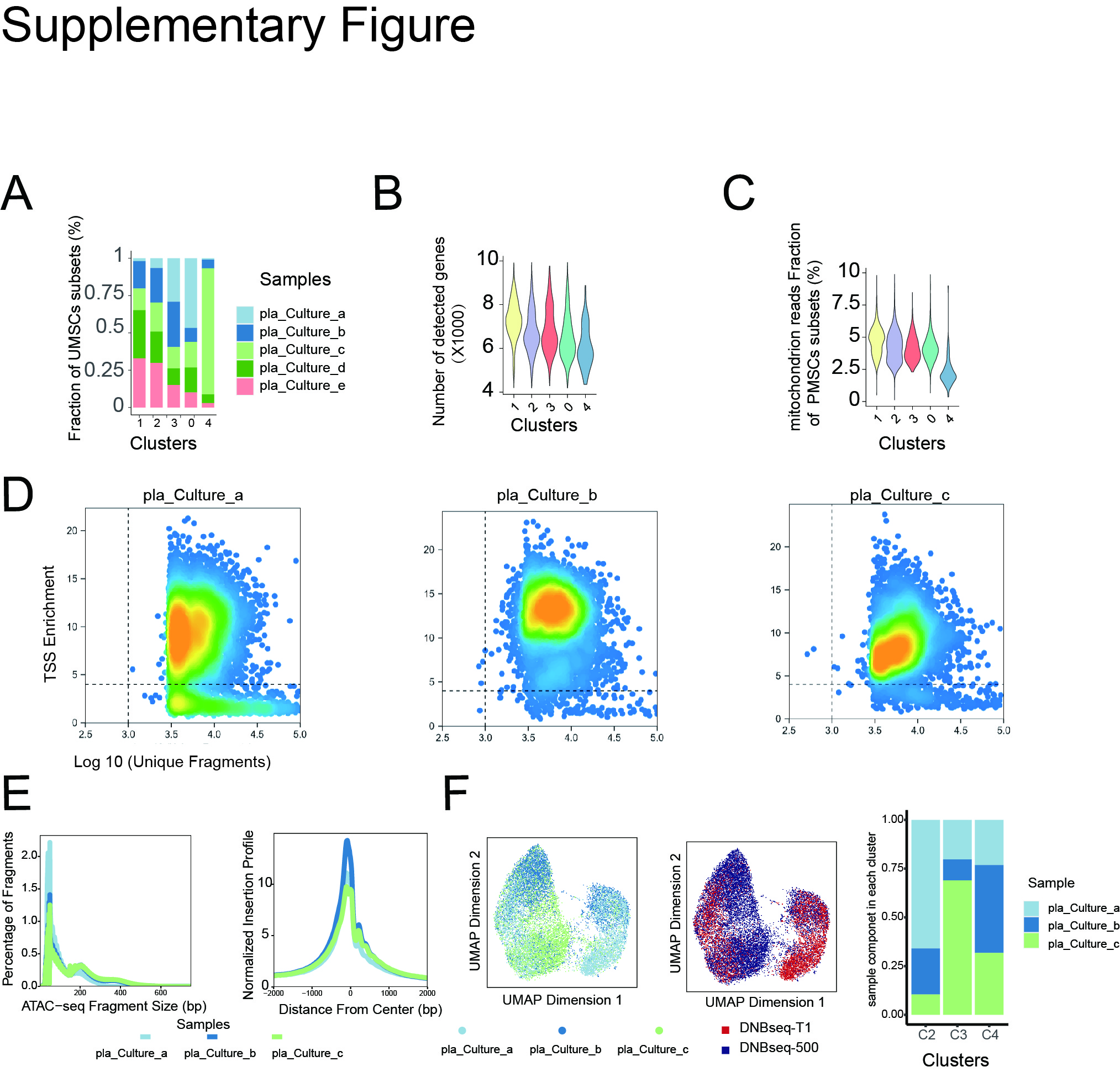

Supplement: Supplementary file 1 [file Image1.jpeg]
