# Supplementary material for: Integrative Single-Cell RNA-Seq and ATAC-Seq Analysis of Mesenchymal Stem/Stromal Cells Derived from Human Placenta
Source: Front Cell Dev Biol. 2022 Apr 5;10:836887. doi: 10.3389/fcell.2022.836887 (PMC9017713; doi:10.3389/fcell.2022.836887)
Supplement: Supplementary file 2 [file DataSheet1.docx]

**Fig 1 Single-Cell Transcriptome Analysis of PMSC**

1. Schematic overview of the workflow. MSCs derived from human placenta were processed for scRNA-seq(n = 5) and scATAC-seq(n = 3).
2. Representative flow cytometric histogram of PMSCs showing the presence of positive MSC markers (CD73, CD90 and CD105) and absence negative MSCs markers (CD34, CD45, CD11b, CD19 and HLA-DR were merged )
3. t-SNE visualization of 31,219 PMSC cells from 5 samples reveals heterogeneous cell states at Single-Cell RNA seq level. Each dot represent a single cell( n=31,219), colored by its corresponding cluster.
4. Bar plot showing the fraction of cell cycle component in each cluster.(bottom)
5. A heatmap shows genes (rows) that are differentially expressed across 5 clusters, colored by relative gene expression (z-score). Glod : high expression; Purple : low expression. Representative gene are highlighted. （p<0.05, logFC>0.25, top90 in each cluster）
6. Boxplot showing the expression level of selected representative DEGs in five clusters.
7. GO terms enrichment of DEGs respective to indicated PMSC clusters.
8. Pseudo temporal developmental trajectory of PMSCs inferred by Monocle.

Bar plot showing the fraction of each cluster component in each branch.(bottom)

1. Immunostaining of MKI67, TOP2A, DEDD2, THY1(CD90), PRDM1, CITED2 and IGFBP6 in PMSCs.

**Fig2 Single-Cell Chromatin Accessibility Analysis of PMSCs**

1. UMAP visualization of 17410 PMSC cells from 3 samples reveals heterogeneous cell states at Single-Cell ATAC seq level. Each dot represent a single cell, colored by its corresponding cluster.
2. The same UMAP visualization shown in Figure 2A but each cell colored by predicted corresponding RNA cell states(n=17356). C1 was removed. (Right)
3. Dendrogram showing relationships among subclusters from scATAC-seq and scRNA-seq.

The variable features of scRNA-seq data identify by Seurat presenting in GeneScoreMatrix and normalized RNA expression matrix were used. The mean score of each gene in each cluster were scaled.

1. Chromatin accessibility for the positive MSC markers (CD73, CD90 and CD105) and negative MSC markers (CD34, PTPRC(CD45), CD19 and HLA-DR).
2. scATAC-seq heatmap of differentially activity gene across 5 clusters, colored by relative gene gene-activity scores (z-score). Gene-activity were converted from accessible peaks calculated in ArchR using Cicero. Glod : high activity; blue: low activity. Representative genes from scRNA-seq and relative function are highlighted. (log2 fold change (LFC) > 0.15 and false discovery rate (FDR)<0.01)
3. UMAP visualization show the gene activity of representative genes select from scRNA-seq in corresponding scATAC-seq clusters.
4. Aggregated scATAC-seq tracks denoting marker chromatin accessibility peaks for each cluster.
5. GO terms enrichment of different activities gene respective to indicated scATAC-seq clusters.

**Fig3 I**[**ntegrated analysis**](javascript:;) **of cell states-specific epigenetic regulators in inferred PMSC subgroups.**

1. Heatmap representing PMSCs cell states marker peaks. Each row represents an individual marker peak, colored by the normalized marker peak accessibility score(Z-score) (Left). Transcription factor motifs and transcription factor and P-value enriched in each cell states marker peak sequences. Transcription factor motifs selected in red.
2. The same UMAP visualization shown in Fig2 A , but each cell colored by the enrichment of TF activity score (deviations) calculated in ArchR using ChromVAR.
3. heatmaps of 13,863 significant peak-to-gene links across cell states (FDR<0.0001; corCutOff > 0.4; varCutOff > 025 when selecting links by plotPeak2GeneHeatmap function) . Top, peak-to-gene links that are identified almost within C4. Middle, peak-to-gene links that are unique to C2. Bottom, peak-to-gene links identified in both C3 and C4,
4. The number of significant peak-gene links for all peaks.
5. The number of significant peak-gene links for all genes.
6. The number of significantly correlated peaks for each gene. Putative DORCs are highlighted.

(G-I) Aggregated scATAC-seq tracks showing genomic regions near *GLRX*(G), *MGLL* (H), and *EIF5A* (I) gene. Differently peaks in cluster are shown in second line. The Loop in third line height represents the significance of peak-to-gene links (corCutOff = 0.45, FDRCutOff = 1e-04, varCutOffATAC = 0.25, varCutOffRNA = 0.25). The RNA expression are present on the left boxplot. The motif enrichment for associated peaks（shown in Figure 3C）are shown in the right and the UMAP show the enrichment of TF activity score (deviations); the boxplot shows the RNA expression of the enriched TF.

**Figure 4 *PRDM1* played crucial role in maintaining immunomodulatory capability of PMSC subgroup.**

1. Aggregated scATAC-seq tracks showing genomic regions near *PRDM1*(Left). the UMAP show the enrichment of *PRDM1* activity score (deviations).
2. Heatmap of different regulon identified by SCENIC.
3. Heatmap of PRDM1 target genes expression(left) and chromatin accibility(right)
4. GO enrichment for *PRDM1* target genes
5. Boxplot showing the expression level of selected representative *PRDM1* target genes *PKIG, CITED2* and *CXCL8.*
6. The UMAP show the enrichment of PRDM1 target genes(*PKIG, CITED2* and *CXCL8*) activity score (deviations).
7. Aggregated scATAC-seq tracks showing genomic regions near *PKIG, CITED2* and *CXCL8*, respectively.
8. Immunostaining of PRDM1 and CXCL8 in PMSCs

**Fig S1. Quality control of scRNA-seq and scATAC-seq datasets.**

1. Bar plot showing the fraction of samples within each scRNA data cluster.
2. Violin plots showing the number of genes (nFeature) in each cluster in scRNA-seq data.
3. Violin plots showing the mitochondrial reads fraction in each cluster in scRNA-seq data.
4. scATAC-seq cell filtering plot of 3 samples. The x-axis is the log transformed unique fragments and the y-axis is enrichment of Tn5 insertions at TSS, representing the robust signal to background for each single cell.
5. The distribution of ATAC-seq fragments in each sample(Left). The enrichment of ATAC-seq reads around TSSs in each sample(Right).
6. The same UMAP visualization shown in Fig2A but each cell colored by Samples (Left) and sequencing platform (Right).
7. Barplot showing the fraction of samples within each scATAC-seq cluster.
